# Supplementary figures and images for: ARDEP, a Rapid Degenerate Primer Design Pipeline Based on k-mers for Amplicon Microbiome Studies
Source: Int J Environ Res Public Health. 2020 Aug 17;17(16):5958. doi: 10.3390/ijerph17165958 (PMC7459862; doi:10.3390/ijerph17165958)

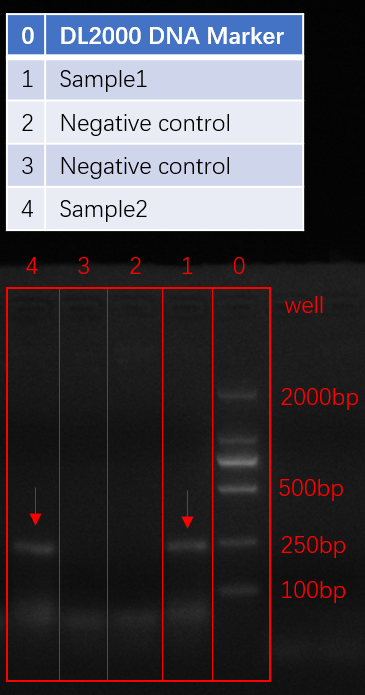

Supplement: Supplementary file 1 [file ijerph-17-05958-s001.zip › Supplementary Materials/Figure S1. Amplification results of primer set AOB_kmer2AOB_kmer28 in soil samples..png]
